# Intestinal Oxalate Absorption, Enteric Hyperoxaluria, and Risk of Urinary Stone Formation in Patients with Crohn’s Disease

**DOI:** 10.3390/nu16020264

**Published:** 2024-01-16

**Authors:** Roswitha Siener, Charlotte Ernsten, Jan Speller, Christian Scheurlen, Tilman Sauerbruch, Albrecht Hesse

**Affiliations:** 1 University Stone Center, Department of Urology, University Hospital Bonn, 53127 Bonn, Germany; s4cherns@uni-bonn.de (C.E.); albrecht-hesse@web.de (A.H.); 2 Department of Medical Biometry, Informatics and Epidemiology, Medical Faculty, University of Bonn, 53127 Bonn, Germany; speller@imbie.uni-bonn.de; 3 Department of Internal Medicine I, University Hospital Bonn, 53127 Bonn, Germany; chris.scheurlen@t-online.de (C.S.); tilman.sauerbruch@ukbonn.de (T.S.)

**Keywords:** urolithiasis, kidney stones, Crohn’s disease, secondary hyperoxaluria, intestinal oxalate absorption, calcium, oxalate, bowel resection, fat malabsorption, diet

## Abstract

Nephrolithiasis is a common urologic manifestation of Crohn’s disease. The purpose of this study was to investigate the clinical characteristics, intestinal oxalate absorption, and risk factors for urinary stone formation in these patients. In total, 27 patients with Crohn’s disease and 27 healthy subjects were included in the present study. Anthropometric, clinical, and 24 h urinary parameters were determined, and the [^13^C_2_]oxalate absorption test was performed. Among all patients, 18 had undergone ileal resection, 9 of whom had a history of urinary stones. Compared to healthy controls, the urinary excretion values of calcium, magnesium, potassium, sulfate, creatinine, and citrate were significantly lower in patients with Crohn’s disease. Intestinal oxalate absorption, the fractional and 24 h urinary oxalate excretion, and the risk of calcium oxalate stone formation were significantly higher in patients with urolithiasis than in patients without urolithiasis or in healthy controls. Regardless of the group, between 83% and 96% of the [^13^C_2_]oxalate was detected in the urine within the first 12 h after ingestion. The length of ileum resection correlated significantly with the intestinal absorption and urinary excretion of oxalate. These findings suggest that enteric hyperoxaluria can be attributed to the hyperabsorption of oxalate following extensive ileal resection. Oral supplementation of calcium and magnesium, as well as alkali citrate therapy, should be considered as treatment options for urolithiasis.

## 1. Introduction

Crohn’s disease, a chronic inflammatory bowel disorder, can affect all segments of the gastrointestinal tract, but most commonly affects the terminal ileum and the colon [1]. Within 20 years after diagnosis, about 50% of patients experience stricturing or penetrating intestinal complications that often require surgery, and the risk of post-operative recurrence is high [2,3,4]. Nephrolithiasis is a common extraintestinal urologic manifestation of Crohn’s disease, with calcium oxalate being the predominant type of stone [5,6,7]. The prevalence of urolithiasis has been reported to be increased in patients after intestinal resection [8,9].

Urinary oxalate excretion is a well-recognized major risk factor for calcium oxalate stone formation [10]. Hyperoxaluria may be attributed to increased endogenous production, high dietary intake, or intestinal hyperabsorption of oxalate [11,12,13,14], or a combination of these pathogenetic factors. Enteric hyperoxaluria, which is frequently observed in patients with ileal resection, is attributed to fat malabsorption and the subsequent increased intestinal oxalate absorption [15,16,17]. Underlying mechanisms that contribute to the hyperabsorption of dietary oxalate and secondary hyperoxaluria include malabsorption of fatty acids that bind calcium, leaving more free oxalate available for absorption, and elevated concentrations of fatty acids and bile salts that cause increased permeability of the colonic mucosa to oxalate [16,18]. Enteric hyperoxaluria is not only associated with an elevated risk of urinary stone formation, especially in patients with intestinal resection [8,9], but can also lead to oxalate nephropathy, which may result in chronic kidney disease and early end-stage renal failure [17,19].

A previous study revealed significantly higher urinary oxalate excretion and intestinal oxalate absorption in pediatric Crohn’s patients with urolithiasis and/or nephrocalcinosis than in adult and pediatric patients, combined, without urolithiasis [20]. However, there is a lack of knowledge about the kinetics of intestinal oxalate absorption and excretion in patients with Crohn’s disease under standardized conditions. Although other urinary abnormalities, aside from hyperoxaluria, may contribute to calcium oxalate stone formation, such as low urine volume and pH, hypomagnesuria, and hypocitraturia, information on the 24 h urine composition and the risk of urinary stone formation in patients with Crohn’s disease is scarce [21,22]. Given the frequency of intestinal resection in Crohn’s disease, little is known about the effects of resection on risk factors for urolithiasis, other than urinary oxalate excretion. The purpose of the present study was, therefore, (1) to investigate the risk profile for urinary stone formation in adult patients with Crohn’s disease compared with healthy controls, (2) to evaluate the effects of the extent of resection on intestinal oxalate absorption and urinary risk factors for stone formation, and (3) to assess the impact of intestinal oxalate absorption on urinary oxalate excretion in patients with Crohn’s disease with or without urolithiasis.

## 2. Materials and Methods

### 2.1. Study Participants

In total, 27 patients, 14 men and 13 women aged 18 to 73 years, with Crohn’s disease were included in the present study. The patients were recruited from the Department of Internal Medicine I and the University Stone Center of the Department of Urology at the University Hospital Bonn. The diagnosis of Crohn’s disease was made through endoscopy/histology and radiology. The Crohn’s disease activity index (CDAI) was determined [23], and the frequency of diarrhea per week was assessed. Patients were asked to maintain their usual dietary habits before their participation in this study.

The control group was to be the same sample size and consisted of 27 healthy individuals, 13 women and 14 men aged 28 to 54 years, attending the University Stone Center of the Department of Urology at the University Hospital Bonn for the [^13^C_2_]oxalate absorption test. The 27 urine samples, from healthy volunteers, were randomly selected. All subjects had normal physical examinations and normal findings with multiparameter urine test strips (Combur9 test, Boehringer, Mannheim, Germany). None of the subjects had a history of bowel disease, urolithiasis, or other significant diseases.

The study participants did not take any medication or dietary supplements during the present study that could influence calcium and oxalate metabolism or acid–base status, such as calcium, vitamin D, steroids, alkali citrate, or sodium bicarbonate. The study was approved by the Ethics Committee of the Medical Faculty of the University of Bonn (8295) and was performed in accordance with the Declaration of Helsinki. All patients and healthy volunteers provided written informed consent prior to this study.

### 2.2. Study Procedure

The medical history, as well as the anthropometric, clinical, and 24 h urinary parameters, were collected from patients with Crohn’s disease at baseline, under their usual, free-choice diet. In the subsequent phase, both patients and healthy controls collected 24 h urine under a balanced, standardized diet. The analysis of 24 h urinary parameters was performed as previously described [24]. The ion activity product indices of calcium oxalate, uric acid, brushite, and struvite were determined [25,26,27]. The relative supersaturationof calcium oxalate, uric acid, brushite, and struvite was calculated using the iterative computer program EQUIL2 [28].

### 2.3. [^13^C_2_]Oxalate Absorption Test

The [^13^C_2_]oxalate absorption test was performed to determine the intestinal oxalate absorption of patients with Crohn’s disease and healthy controls [29,30]. The test was conducted on two consecutive days under controlled, standardized dietary conditions. The first day (day 1) was designed to achieve a steady state in order to enable an adequate calibration procedure. On the first day, the study participants collected a 24 h blank urine in two 12 h portions. The second day (day 2) was scheduled for the main test phase. On the morning of the second day at 8 a.m., the study participants were orally administered a capsule containing 50 mg sodium [^13^C_2_]oxalate, corresponding to 33.8 mg [^13^C_2_]oxalic acid, with 100 mL water. On the second day, urine was completely collected from 8 a.m. to 2 p.m. (fraction 1; 6 h), 2 p.m. to 8 p.m. (fraction 2; 6 h) and 8 p.m. to 8 a.m. (fraction 3; 12 h). All storage bottles contained 25% hydrochloric acid as a preservative to prevent bacterial growth and the precipitation of calcium oxalate. Aliquots of urine samples were stored at −20 °C. The subsequent quantitative determination of [^13^C_2_]oxalate in the urine fractions from the second day was carried out using gas chromatography–mass spectrometry, as previously described in detail [29]. Intestinal oxalate absorption was calculated from urinary [^13^C_2_]oxalate and expressed as a percentage of the orally administered dose of [^13^C_2_]oxalate. Hyperabsorption of oxalate is defined as intestinal absorption greater than 10% [12,31].

### 2.4. Statistical Analysis

The non-parametric Mann–Whitney U test for unpaired data was used to compare continuous variables between two groups. Categorical variables were compared using Fisher’s exact test. Correlations between variables were calculated using Spearman’s rank correlation. All statistical tests were two-sided for the exclusively explorative analysis, with a significance level α = 0.05, without taking into account the effects of multiple testing. Statistical analyses were performed using IBM SPSS for Windows versions 27.0 and 28.0 (SPSS Inc., Chicago, IL, USA).

## 3. Results

### 3.1. Characteristics of Study Participants

The baseline characteristics of the 27 patients with Crohn’s disease are presented in Table 1. Among all patients, 18 patients, 10 women and 8 men, had undergone ileal resection. The length of ileum resected varied from 15 to 120 cm, according to the surgical reports. While the CDAI did not differ between patients with and without ileal resection, patients with ileal resection were significantly older than those without (46.7 ± 10.9 versus 28.9 ± 9.8 years, respectively; *p* = 0.001), and they were more frequently affected by diarrhea than patients without ileal resection (83% versus 33%, respectively; *p* = 0.026).

Nine patients with Crohn’s disease had a history of calcium oxalate urolithiasis. All patients with urinary stone disease had already undergone ileal resection, compared with 50% of patients without urolithiasis. The median length of the ileum resected was 65 cm (range: 37 to 120 cm) in patients with urolithiasis and 27 cm (range: 15 to 56 cm) in patients without a history of urinary stone disease and, thus, differed significantly between the two groups (*p* = 0.002).

### 3.2. Urine Composition

The 24 h urine composition of patients with Crohn’s disease under their usual, free-choice diet is shown in Table 2. Urinary pH, calcium, magnesium, sulfate, uric acid, and citrate excretion were significantly lower in patients with ileal resection than in patients without resection. The relative supersaturation levels of brushite and struvite were significantly lower, and the relative supersaturation of uric acid was significantly higher in patients with ileal resection than in patients without ileal resection. The ion activity product index and the relative supersaturation of calcium oxalate were similar in both groups. No statistically significant difference was observed in the ion activity product index of brushite, nor in the urinary excretion of other parameters, including oxalate.

In patients with Crohn’s disease and urolithiasis, urinary oxalate excretion, the relative supersaturation of calcium oxalate, and the ion activity product index of calcium oxalate were significantly higher compared to patients without urolithiasis. For all other urinary parameters, no statistically significant differences were found between patients with Crohn’s disease with and without urolithiasis.

The 24 h urine composition of patients with Crohn’s disease and healthy controls under standardized conditions is presented in Table 3. The urinary excretion values of potassium, calcium, magnesium, sulfate, creatinine, and citrate were significantly lower in patients with Crohn’s disease than in healthy controls. Urinary calcium and sulfate excretion were significantly lower in patients with ileal resection than in patients without ileal resection. Mean urinary oxalate excretion was significantly higher in patients with Crohn’s disease and urolithiasis than in patients without stone disease. While urinary density, sodium, potassium, calcium, magnesium, sulfate, and citrate excretion were significantly lower in patients with Crohn’s disease and urolithiasis than in healthy controls, urinary oxalate excretion and the ion activity product index of calcium oxalate were significantly higher. No statistically significant differences were observed between the groups for any other urinary parameters.

### 3.3. Intestinal [^13^C_2_]Oxalate Absorption

The results of the [^13^C_2_]oxalate absorption test are presented in Table 4. Intestinal [^13^C_2_]oxalate absorption was significantly higher in patients with Crohn’s disease with urolithiasis than in both patients without stone disease and healthy controls. Intestinal hyperabsorption of oxalate, defined as oxalate absorption ≥ 10%, was diagnosed in 100% of patients with Crohn’s disease and urolithiasis. Except for the labeled urinary oxalate excretion in fraction 1, the labeled, unlabeled, and total oxalate excretion on the test day of the [^13^C_2_]oxalate absorption test were significantly higher in patients with Crohn’s disease with urolithiasis than in both patients without urolithiasis and healthy controls. Intestinal [^13^C_2_]oxalate absorption and the urinary excretion of labeled, unlabeled, and total oxalate did not differ between patients with and without ileal resection. In healthy individuals, the unlabeled urinary oxalate excretion in fraction 1, labeled oxalate excretion in fraction 2 and 3, and total urinary oxalate excretion in fraction 1 were significantly lower than in patients with Crohn’s disease. Intestinal [^13^C_2_]oxalate absorption, as well as the urinary excretion of total labeled, total unlabeled, and the sum of total labeled and unlabeled oxalate did not differ between patients with Crohn’s disease and healthy controls. In each group, most of the [^13^C_2_]oxalate absorbed by the intestine, i.e., between 83% and 96%, was detected in the urine within the first 12 h after ingestion.

**Figure 1:**
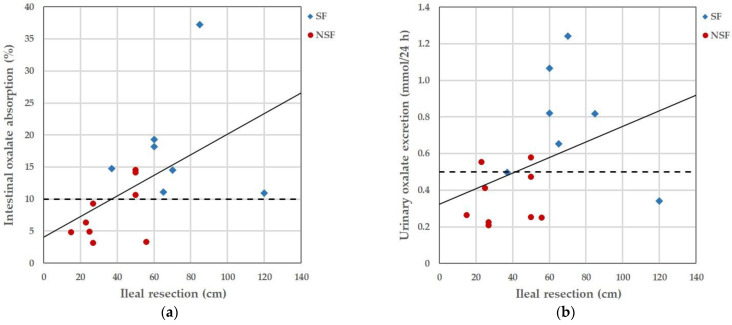
Correlation between the length of the ileal resection and intestinal oxalate absorption, and between the length of the ileal resection and urinary oxalate excretion under standardized dietary conditions: (**a**) length of ileal resection and intestinal oxalate absorption and (**b**) length of ileal resection and urinary oxalate excretion. The solid line shows the univariate linear regression. The dashed lines present the reference values for intestinal oxalate absorption and hyperoxaluria, respectively. Abbreviations: SF, stone formers; NSF, non-stone formers.

Intestinal [^13^C_2_]oxalate absorption correlated significantly positively with urinary oxalate excretion, both in patients with Crohn’s disease (R = 0.545; *p* = 0.003) and in healthy individuals under standardized conditions (R = 0.450; *p* = 0.019). No monotonic correlations were detected between intestinal [^13^C_2_]oxalate absorption and clinical signs of Crohn’s disease, as assessed with the CDAI (R = 0.147; *p* = 0.504), or the frequency of diarrhea (R = 0.350; *p* = 0.073). Among patients with Crohn’s disease after ileal resection, a positive correlation was observed between the length of the ileum resection and intestinal oxalate absorption (R = 0.609, *p* = 0.012) (Figure 1a), between the length of resection and urinary oxalate excretion (R = 0.520; *p* = 0.039) (Figure 1b), and between intestinal oxalate absorption and urinary oxalate excretion, under standardized conditions (R = 0.658; *p* = 0.003). 

## 4. Discussion

Urolithiasis is a common urologic manifestation of Crohn’s disease [5,6]. Several stone-forming abnormalities have been reported to favor calcium oxalate stone formation in patients with Crohn’s disease, including low urine volume, hypomagnesuria, hypocitraturia, and hyperoxaluria [21,22,32,33]. In the present study, the comparison of the 24 h urine composition of patients with Crohn’s disease and healthy controls under standardized dietary conditions revealed significantly lower urinary excretion values of calcium, magnesium, potassium, and citrate in patients with Crohn’s disease, while urinary volume and oxalate excretion were similar in both groups. Despite the significantly lower excretion of the urinary inhibitors magnesium and citrate, no difference was observed in the ion activity product index of calcium oxalate, which is probably due to the simultaneously lower urinary calcium excretion in patients with Crohn’s disease. Hypomagnesuria is presumably due to the malabsorption of magnesium from the diet, whereas hypocitraturia appears to be mainly the result of a loss of bicarbonate in the feces that is associated with chronic diarrhea, often with subsequent metabolic acidosis [34,35,36]. The nutritional status of patients with inflammatory bowel disease is often impaired, with malnutrition presenting as imbalanced energy or nutrient intake, including protein–energy malnutrition, disease-related malnutrition, sarcopenia, and micronutrient deficiency [37]. The pathogenesis of malnutrition involves several factors, such as reduced food intake, intestinal malabsorption, chronic loss of protein in stool, and increased energy requirements due to hypercatabolism [37,38,39]. In the present study, urinary excretion of sulfate and creatinine were significantly lower in patients with Crohn’s disease than in healthy controls. Since the majority of urinary sulfate is derived from sulfur-containing amino acids, sulfate excretion is a suitable biomarker of dietary intake and metabolism of protein [40,41]. Moreover, urinary creatinine excretion reflects muscle mass, as creatinine is a waste product of muscle metabolism [42,43]. It is hypothesized that restricted dietary protein intake and/or chronic loss of protein in stool might have contributed to the lower muscle mass in patients with Crohn’s disease in the present study, as determined from 24 h urinary creatinine excretion. A systematic review revealed a prevalence of sarcopenia of 42% in Crohn’s disease [44].

Several studies reported that the risk of urolithiasis increases after intestinal resection, which often has to be performed in Crohn’s disease [35,45]. In the present study, urinary calcium and sulfate excretion were significantly lower in patients with ileal resection than in patients without ileal resection, under both their usual and the standardized dietary conditions. No statistically significant difference was observed between the groups in intestinal oxalate absorption. Urinary oxalate excretion and the ion activity product index did not differ between the two groups, under neither their usual nor the standardized dietary conditions. Moreover, the relative supersaturation of calcium oxalate was similar between both groups under their usual diets. The findings of this study indicate that ileal resection is not, per se, the decisive determinant of calcium oxalate stone formation in patients with Crohn’s disease.

A previous study suggested that the prevalence of urinary stone disease is associated with the length of bowel resection [8]. It has been assumed that the localization and length of intestinal resection have significant impacts on the extent of the intestinal absorption and urinary excretion of oxalate [18,32,46,47]. A study of a population-based cohort of patients with Crohn’s disease reported that the median cumulative length of total bowel resected was 64 cm (IQR, 38–93) during the follow-up period, 36 cm (17–60) for small bowel resected, and 15 cm (8–50) for colon resected [48]. In the present study, all patients with Crohn’s disease and urolithiasis had undergone ileal resection, compared with only 50% of patients without urinary stone disease. In patients with a history of urinary stone formation, the median length of ileum resected was 65 cm and was more than twice the length of the ileum resected in patients without a history of urinary stone disease (27 cm). Mean urinary oxalate excretion and the risk of calcium oxalate stone formation were extraordinarily elevated and significantly higher in patients with Crohn’s disease and urolithiasis than in patients without a history of urinary stone disease under their usual dietary conditions. Moreover, the fractional and 24 h urinary excretion of oxalate were significantly higher in patients with urolithiasis than in either patients without urolithiasis or healthy subjects under standardized dietary conditions. Enteric hyperoxaluria was present in 67% of patients with urolithiasis under controlled standardized conditions. Among patients with Crohn’s disease and ileum resection, a positive correlation was observed between the length of resection and urinary oxalate excretion. Apart from strongly elevated urinary oxalate excretion, no differences in other lithogenic or inhibitory urinary parameters were observed in patients with Crohn’s disease with and without urolithiasis. Hyperoxaluria is an important pathogenic factor in calcium oxalate stone formation in patients with gastrointestinal disorders associated with fat malabsorption [18,20,49,50,51]. The present study revealed that enteric hyperoxaluria can be attributed a pivotal role in urinary stone formation in patients with Crohn’s disease. It is concluded that enteric hyperoxaluria due to elevated fractional and 24 h urinary oxalate, associated with extensive ileal resection, is the main determinant of urinary stone formation in patients with Crohn’s disease.

Hyperoxaluria in patients with Crohn’s disease can result from increased intestinal oxalate absorption. Previous studies using the [^13^C_2_]oxalate absorption test already revealed an association between the intestinal absorption and urinary excretion of oxalate, but only in idiopathic calcium oxalate stone formers [12], or in a combined group of pediatric and adult patients with Crohn’s disease [20]. Using the [^13^C_2_]oxalate absorption test, the present study revealed that intestinal oxalate absorption was significantly higher in adult patients with Crohn’s disease and urolithiasis than in patients without a history of urinary stone formation or healthy subjects. Moreover, a positive correlation was observed between the length of ileal resection and intestinal oxalate absorption, and between intestinal oxalate absorption and urinary oxalate excretion. Intestinal hyperabsorption of oxalate, defined as oxalate absorption ≥ 10%, was diagnosed in all patients with Crohn’s disease and urolithiasis. Regardless of the group, i.e., patients with Crohn’s disease with or without urolithiasis, with or without ileum resection, and healthy controls, most of the [^13^C_2_]oxalate absorbed by the intestine, i.e., between 83% and 96%, was detected in the urine within the first 12 h after ingestion. To our knowledge, the current study is the first study to investigate the fractional intestinal absorption of oxalate in patients with Crohn’s disease under controlled, standardized conditions. However, urinary oxalate excretion in patients with urolithiasis was significantly higher in all but one fraction than in patients without a history of stone disease. Mechanisms that contribute to the increased intestinal hyperabsorption of oxalate and enteric hyperoxaluria in patients with Crohn’s disease and urolithiasis include the malabsorption of free fatty acids in the small intestine that bind dietary calcium, leaving more free oxalate for absorption, and elevated permeability of the colonic mucosa caused by increased concentrations of bile salts and fatty acids [15,16,18,52].

Another potential factor in the pathophysiology of hyperoxaluria is the gut microbiome. Colonization with *Oxalobacter formigenes*, a Gram-negative anaerobic bacterium that can metabolize dietary oxalate in the human colon, was found to be less frequent in patients with both inflammatory bowel disease and calcium oxalate urolithiasis compared to healthy controls [53]. However, the majority of these patients with inflammatory bowel disease had ulcerative colitis, which is not considered a risk for enteric hyperoxaluria. The role of the lack of intestinal colonization with oxalate-degrading bacteria in the development of enteric hyperoxaluria in patients with Crohn’s disease remains to be clarified.

Dietary intervention is an integral part of the treatment of patients with Crohn’s disease and urolithiasis. A cornerstone of dietary therapy for enteric hyperoxaluria is the restriction of dietary oxalate intake [25,36]. Dietary oxalate is mainly contained in plant foods [54]. Although vegetable and fruit juices can be beneficial in the recurrence prevention of calcium oxalate stone disease, due to their alkalizing properties, their oxalate contents must be taken into account [55]. Detailed information about the oxalate contents of beverages and foods is the essential basis for the treatment of patients with enteric hyperoxaluria. Unfortunately, data on the dietary oxalate intake of the participants in the present study were not available. However, the [^13^C_2_]oxalate absorption test revealed an intestinal oxalate absorption of 7.2% in healthy subjects compared to 17.4% in patients with Crohn’s disease and urolithiasis under standardized dietary conditions. Both calcium and magnesium have the potential to bind oxalate in the intestine and, thus, reduce the absorption and urinary excretion of oxalate [56,57]. Oral supplementation of calcium, in patients with low urinary calcium excretion, and magnesium should, therefore, be considered as therapeutic measures for patients with enteric hyperoxaluria [25]. Although the oral administration of oxalate decarboxylase, an oxalate-degrading enzyme, resulted in a modest reduction in urinary oxalate excretion in patients with enteric hyperoxaluria, larger studies of longer durations are needed to evaluate the potential impacts on clinical outcomes [58]. Due to the significantly lower potassium and citrate excretion in patients with Crohn’s disease than in healthy controls under standardized dietary conditions, patients with Crohn’s disease may benefit from oral alkali therapy [17,25]. Overall, however, treatment options for patients with Crohn’s disease and urolithiasis remain limited.

## 5. Conclusions

Urolithiasis in patients with Crohn’s disease is attributed to secondary hyperoxaluria. The findings of this study suggest that enteric hyperoxaluria is primarily due to the hyperabsorption of oxalate following extensive ileal resection. The length of ileal resection was found to be the main determinant of urinary stone formation in patients with Crohn’s disease. Dietary measures for enteric hyperoxaluria are limited to restricting dietary oxalate intake, as well as oral supplementation of calcium and magnesium. Patients with Crohn’s disease may also benefit from oral alkali therapy. While treatment options for enteric hyperoxaluria are still limited, it should be considered that maximal preservation of the intestine reduces the risk of urinary stone formation. The nutritional status of patients with Crohn’s disease should be monitored to avoid malnutrition, including sarcopenia and mineral deficiencies. Novel therapeutic strategies are needed to prevent complications of Crohn’s disease that require surgery, as well as to mitigate the consequences of enteric hyperoxaluria.

## Figures and Tables

**Table 1:** Characteristics of patients with Crohn’s disease.

	CD Patients Total	CD Patients with IR	CD Patients without IR		CD Patients with UL	CD Patients without UL	
	n = 27	n = 18	n = 9		n = 9	n = 18	
	Mean ± SD n (%)	Mean ± SD n (%)	Mean ± SD n (%)	*p* Value ^a^	Mean ± SD n (%)	Mean ± SD n (%)	*p* Value ^b^
Women	13 (48.1%)	10 (55.6%)	3 (33.3%)	0.420	5 (55.6%)	8 (44.4%)	0.695
Men	14 (51.9%)	8 (44.4%)	6 (66.7%)		4 (44.4%)	10 (55.6%)	
Age (years)	40.8 ± 13.4	46.7 ± 10.9	28.9 ± 9.8	0.001	44.4 ± 8.2	38.9 ± 15.3	0.269
Weight (kg)	67.0 ± 14.4	67.4 ± 16.1	66.2 ± 11.0	0.990	65.2 ± 14.6	67.9 ± 14.6	0.658
Height (m)	1.73 ± 0.09	1.71 ± 0.09	1.76 ± 0.08	0.216	1.72 ± 0.08	1.73 ± 0.09	0.604
BMI (kg/m²)	22.4 ± 4.2	22.9 ± 4.4	21.5 ± 3.9	0.375	22.0 ± 4.0	22.6 ± 4.4	0.860
Smokers	12 (44.4%)	9 (50.0%)	3 (33.3%)	0.683	5 (55.6%)	7 (38.9%)	0.448
CDAI	148 ± 110 ^c^	173 ± 107 ^d^	108 ± 108	0.201	177 ± 74 ^e^	140 ± 119	0.363
Diarrhea (n)	18 (66.7%)	15 (83.3%)	3 (33.3%)	0.026	8 (88.9%)	10 (55.6%)	0.193
Ileal resection (n)	18 (66.7%)	18 (100%)	0 (0%)	-	9 (100.0%)	9 (50.0%)	0.012
Length of ileum resection (cm)	51 ± 27 ^f^	51 ± 27 ^f^	-	-	71 ± 26 ^g^	36 ± 15 ^h^	0.002
Colon resection (n)	11/22 (50.0%) ^i^	11/13 (84.6%) ^i^	0 (0%)	<0.001 ^j^	6/8 (75.0%) ^k^	5/14 (35.7%) ^l^	0.183
Length of colon resection (cm)	22 ± 16 ^m^	22 ± 16 ^m^	-	-	25 ± 10 ^n^	19 ± 20 ^l^	0.262
Urolithiasis (n)	9 (33.3%)	9 (50.0%)	0 (0%)	0.012 ^j^	9 (100%)	0 (0%)	-

Abbreviations: CD, Crohn’s disease; CDAI, Crohn’s disease activity index; IR, ileal resection; SD, standard deviation; UL, urolithiasis. ^a^ *p*-value: CD patients with IR vs. CD patients without IR; ^b^ *p*-value: CD patients with UL vs. CD patients without UL; ^c^ n = 23 (10 women, 13 men); ^d^ n = 14 (7 women, 7 men); ^e^ n = 5 (2 women, 3 men); ^f^ n = 16 (9 women, 7 men); ^g^ n = 7 (4 women, 3 men); ^h^ n = 9 (5 women, 4 men); ^i^ n = 11 (5 women, 6 men); ^j^ category with zero entries; ^k^ n = 6 (4 women, 2 men); ^l^ n = 5 (1 woman, 4 men); ^m^ n = 9 (3 women, 6 men); ^n^ n = 4 (2 women, 2 men).

**Table 2:** Urinary parameters of patients with Crohn’s disease under usual dietary conditions.

	CD Patients Total	CD Patients with IR	CD Patients without IR		CD Patients with UL	CD Patients without UL	
	n = 27	n = 18	n = 9		n = 9	n = 18	
	Mean ± SD	Mean ± SD	Mean ± SD	*p* Value ^a^	Mean ± SD	Mean ± SD	*p* Value ^b^
Volume (L/24 h)	2.063 ± 1.027	1.824 ± 0.810	2.539 ± 1.285	0.067	1.762 ± 0.560	2.213 ± 1.181	0.403
Density (g/cm³)	1.011 ± 0.004	1.010 ± 0.003	1.013 ± 0.005	0.325	1.011 ± 0.004	1.011 ± 0.004	0.789
Urinary pH	5.91 ± 0.46	5.69 ± 0.31	6.35 ± 0.38	<0.001	5.68 ± 0.34	6.03 ± 0.47	0.078
Sodium (mmol/24 h)	194 ± 111	165 ± 91	252 ± 131	0.097	170 ± 86	207 ± 123	0.604
Potassium (mmol/24 h)	47 ± 25	38 ± 13	64 ± 34	0.065	36 ± 11	52 ± 28	0.180
Calcium (mmol/24 h)	3.73 ± 2.88	2.62 ± 1.61	5.95 ± 3.60	0.023	3.22 ± 1.53	3.98 ± 3.37	0.820
Magnesium (mmol/24 h)	2.65 ± 1.69	2.17 ± 1.59	3.63 ± 1.54	0.012	2.11 ± 1.09	2.92 ± 1.90	0.269
Ammonium (mmol/24 h)	42.5 ± 22.5	45.7 ± 25.0	36.2 ± 16.0	0.382	48.7 ± 25.5	39.5 ± 21.0	0.368
Chloride (mmol/24 h)	204 ± 104	184 ± 95	243 ± 114	0.106	188 ± 79	212 ± 115	0.596
Phosphate (mmol/24 h)	25.5 ± 9.1	24.9 ± 8.9	26.7 ± 9.8	0.631	28.4 ± 9.5	24.1 ± 8.8	0.322
Sulfate (mmol/24 h)	16.1 ± 7.5	12.9 ± 5.5	22.3 ± 7.3	0.002	13.8 ± 6.1	17.2 ± 8.0	0.382
Creatinine (mmol/24 h)	12.05 ± 4.29	11.84 ± 4.94	12.47 ± 2.77	0.470	13.19 ± 5.28	11.47 ± 3.75	0.659
Uric acid (mmol/24 h)	3.26 ± 1.44	2.90 ± 1.42	3.98 ± 1.26	0.015	3.29 ± 1.54	3.25 ± 1.43	0.715
Oxalate (mmol/24 h)	0.477 ± 0.230	0.501 ± 0.256	0.428 ± 0.167	0.561	0.623 ± 0.236	0.404 ± 0.194	0.027
Citrate (mmol/24 h)	1.148 ± 1.285	0.821 ± 1.007	1.801 ± 1.579	0.046	0.661 ± 1.038	1.391 ± 1.353	0.076
AP Brushite index	4.01 ± 3.70	3.34 ± 3.75	5.33 ± 3.43	0.118	4.71 ± 4.34	3.65 ± 3.42	0.375
AP Struvite index	2.27 ± 4.85	0.65 ± 1.25	5.49 ± 7.46	<0.001	0.65 ± 0.87	3.07 ± 5.79	0.212
AP Uric acid (10^−9^)	1.25 ± 1.11	1.55 ± 1.08	0.67 ± 0.97	0.009	1.77 ± 1.30	1.00 ± 0.94	0.085
AP Calcium oxalate index	1.41 ± 1.04	1.50 ± 1.20	1.23 ± 0.66	0.940	2.16 ± 1.11	1.04 ± 0.81	0.003
RS Brushite	0.562 ± 0.545	0.327 ± 0.365	1.033 ± 0.555	0.002	0.403 ± 0.347	0.641 ± 0.615	0.980
RS Struvite	0.029 ± 0.052	0.012 ± 0.020	0.065 ± 0.076	<0.001	0.012 ± 0.014	0.038 ± 0.062	0.253
RS Uric acid	1.807 ± 1.506	2.210 ± 1.435	1.001 ± 1.377	0.009	2.509 ± 1.695	1.456 ± 1.313	0.076
RS Calcium oxalate	5.813 ± 3.787	6.120 ± 4.222	5.199 ± 2.842	0.820	8.481 ± 3.413	4.479 ± 3.285	0.005

Abbreviations: AP, ion activity product; CD, Crohn’s disease; IR, ileal resection; RS, relative supersaturation; SD, standard deviation; UL, urolithiasis. ^a^ *p*-value: CD patients with IR vs. CD patients without IR; ^b^ *p*-value: CD patients with UL vs. CD patients without UL.

**Table 3:** Urinary parameters of patients with Crohn’s disease and healthy subjects under standardized dietary conditions.

	CD Patients Total	Healthy Controls		CD Patients with IR	CD Patients without IR		CD Patients with UL	CD Patients without UL		
	n = 27	n = 27		n = 18	n = 9		n = 9	n = 18		
	Mean ± SD	Mean ± SD	*p* Value ^a^	Mean ± SD	Mean ± SD	*p* Value ^b^	Mean ± SD	Mean ± SD	*p* Value ^c^	*p* Value ^d^
Volume (L/24 h)	2.192 ± 0.985	2.142 ± 0.668	0.945	2.027 ± 0.773	2.522 ± 1.303	0.375	2.324 ± 0.791	2.126 ± 1.084	0.463	0.641
Density (g/cm^3^)	1.013 ± 0.006	1.016 ± 0.006	0.095	1.013 ± 0.005	1.015 ± 0.008	0.501	1.011 ± 0.005	1.014 ± 0.006	0.154	0.027
Sodium (mmol/24 h)	167 ± 98	182 ± 45	0.093	140 ± 77	222 ± 117	0.061	143 ± 85	180 ± 104	0.316	0.030
Potassium (mmol/24 h)	45 ± 21	76 ± 23	<0.001	41 ± 16	54 ± 27	0.280	43 ± 16	47 ± 23	0.950	0.001
Calcium (mmol/24 h)	3.36 ± 2.41	4.42 ± 2.16	0.026	2.46 ± 1.41	5.18 ± 3.03	0.007	2.90 ± 1.32	3.60 ± 2.81	0.820	0.035
Magnesium (mmol/24 h)	2.58 ± 1.57	5.22 ± 1.57	<0.001	2.19 ± 1.38	3.35 ± 1.73	0.109	2.18 ± 1.05	2.78 ± 1.77	0.622	<0.001
Phosphate (mmol/24 h)	23.6 ± 8.1 ^e^	26.4 ± 8.7	0.499	23.0 ± 6.6	24.8 ± 11.1 ^f^	0.595	26.5 ± 4.8	22.0 ± 9.1 ^g^	0.177	0.699
Sulfate (mmol/24 h)	15.4 ± 6.6	20.9 ± 4.9	0.001	12.6 ± 4.7	21.0 ± 6.4	0.002	13.2 ± 2.5	16.5 ± 7.8	0.322	<0.001
Creatinine (mmol/24 h)	10.88 ± 3.26	13.89 ± 3.34	0.002	10.96 ± 3.66	10.74 ± 2.47	0.930	11.80 ± 3.52	10.43 ± 3.13	0.440	0.067
Oxalate (mmol/24 h)	0.468 ± 0.260	0.383 ± 0.087	0.595	0.531 ± 0.296	0.341 ± 0.078	0.131	0.706 ± 0.309	0.349 ± 0.115	0.001	<0.001
Citrate (mmol/24 h)	1.107 ± 0.964	3.968 ± 1.486	<0.001	0.969 ± 0.926	1.385 ± 1.034	0.160	0.766 ± 0.679	1.278 ± 1.055	0.131	<0.001
AP CaOx index	1.11 ± 0.89	0.85 ± 0.61	0.138	1.17 ± 1.02	1.01 ± 0.60	1.000	1.60 ± 1.20	0.87 ± 0.59	0.076	0.014

Abbreviations: AP CaOx, ion activity product of calcium oxalate; CD, Crohn’s disease; IR, ileal resection; SD, standard deviation; UL, urolithiasis. ^a^ *p*-value: CD patients vs. healthy controls; ^b^ *p*-value: CD patients with IR vs. CD patients without IR; ^c^ *p*-value: CD patients with UL vs. CD patients without UL; ^d^ *p*-value: CD patients with UL vs. healthy controls; ^e^ n = 26 (13 women, 13 men); ^f^ n = 8 (3 women, 5 men); ^g^ n = 17 (8 women, 9 men).

**Table 4:** Intestinal oxalate absorption, as well as fractional and 24 h urinary excretion of labeled ([^13^C_2_]oxalate), unlabeled, and total oxalate of patients with Crohn’s disease and healthy subjects under standardized dietary conditions.

	CD Patients Total	Healthy Controls		CD Patients with IR	CD Patients without IR		CD Patients with UL	CD Patients without UL		
	n = 27	n = 27		n = 18	n = 9		n = 9	n = 18		
	Mean ± SD	Mean ± SD	*p* Value ^a^	Mean ± SD	Mean ± SD	*p* Value ^b^	Mean ± SD	Mean ± SD	*p* Value ^c^	*p* Value ^d^
Unlabeled urinary oxalate (mmol/6 h) Fraction 1	0.112 ± 0.059	0.077 ± 0.034	0.009	0.116 ± 0.057	0.103 ± 0.064	0.410	0.149 ± 0.055	0.093 ± 0.052	0.007	<0.001
Unlabeled urinary oxalate (mmol/6 h) Fraction 2	0.139 ± 0.110	0.103 ± 0.053	0.272	0.155 ± 0.130	0.106 ± 0.036	0.527	0.215 ± 0.157	0.100 ± 0.047	0.012	0.009
Unlabeled urinary oxalate (mmol/12 h) Fraction 3	0.231 ± 0.147	0.175 ± 0.050	0.258	0.264 ± 0.163	0.166 ± 0.081	0.131	0.306 ± 0.134	0.194 ± 0.141	0.020	0.002
Total unlabeled oxalate (mmol/24 h)	0.481 ± 0.278	0.354 ± 0.096	0.148	0.535 ± 0.309	0.375 ± 0.168	0.189	0.670 ± 0.317	0.387 ± 0.206	0.005	<0.001
[^13^C_2_]oxalate (mmol/6 h) Fraction 1	0.023 ± 0.014	0.022 ± 0.014	0.715	0.024 ± 0.013	0.021 ± 0.015	0.502	0.030 ± 0.014	0.020 ± 0.012	0.058	0.134
[^13^C_2_]oxalate (mmol/6 h) Fraction 2	0.016 ± 0.028	0.004 ± 0.003	0.001	0.018 ± 0.030	0.013 ± 0.024	0.073	0.028 ± 0.040	0.010 ± 0.017	0.010	<0.001
[^13^C_2_]oxalate (mmol/12 h) Fraction 3	0.005 ± 0.012	0.001 ± 0.002	0.046	0.005 ± 0.006	0.007 ± 0.019	0.162	0.007 ± 0.007	0.004 ± 0.014	0.003	<0.001
Total [^13^C_2_]oxalate (mmol/24 h)	0.045 ± 0.039	0.027 ± 0.016	0.064	0.047 ± 0.031	0.041 ± 0.053	0.131	0.065 ± 0.033	0.035 ± 0.038	0.002	<0.001
Total urinary oxalate (mmol/6 h) Fraction 1	0.135 ± 0.067	0.099 ± 0.044	0.024	0.140 ± 0.065	0.124 ± 0.074	0.433	0.179 ± 0.059	0.113 ± 0.061	0.009	<0.001
Total urinary oxalate (mmol/6 h) Fraction 2	0.155 ± 0.121	0.107 ± 0.054	0.162	0.173 ± 0.141	0.119 ± 0.057	0.463	0.243 ± 0.167	0.111 ± 0.056	0.007	0.004
Total urinary oxalate (mmol/12 h) Fraction 3	0.237 ± 0.150	0.176 ± 0.051	0.221	0.269 ± 0.165	0.173 ± 0.093	0.131	0.314 ± 0.136	0.198 ± 0.145	0.020	0.001
Total urinary oxalate (mmol/24 h)	0.526 ± 0.295	0.381 ± 0.104	0.133	0.582 ± 0.320	0.416 ± 0.211	0.176	0.736 ± 0.318	0.422 ± 0.225	0.003	<0.001
										
Intestinal oxalate absorption (%)	12.1 ± 10.2	7.2 ± 4.2	0.055	12.6 ± 8.0	10.9 ± 14.1	0.128	17.4 ± 8.1	9.4 ± 10.3	0.002	<0.001

Abbreviations: CD, Crohn’s disease; IR, ileal resection; SD, standard deviation; UL, urolithiasis. ^a^ *p*-value: CD patients vs. healthy controls; ^b^ *p*-value: CD patients with IR vs. CD patients without IR; ^c^ *p*-value: CD patients with UL vs. CD patients without UL; ^d^ *p*-value: CD patients with UL vs. healthy controls.

## Data Availability

The data presented in this study are available upon reasonable personal request.

## References

[1] Torres J., Mehandru S., Colombel J.-F., Peyrin-Biroulet L. (2017). Crohn’s disease. Lancet.

[2] Thia K.T., Sandborn W.J., Harmsen W.S., Zinsmeister A.R., Loftus E.V. (2010). Risk factors associated with progression to intestinal complications of Crohn’s disease in a population-based cohort. Gastroenterology.

[3] Peyrin-Biroulet L., Loftus E.V., Colombel J.-F., Sandborn W.J. (2010). The natural history of adult Crohn’s disease in population-based cohorts. Am. J. Gastroenterol..

[4] Le Berre C., Danese S., Peyrin-Biroulet L. (2023). Can we change the natural course of inflammatory bowel disease?. Therap. Adv. Gastroenterol..

[5] Greenstein A.J., Janowitz H.D., Sachar D.B. (1976). The extra-intestinal complications of Crohn’s disease and ulcerative colitis: A study of 700 patients. Medicine.

[6] Manganiotis A.N., Banner M.P., Malkowicz S.B. (2001). Urologic complications of Crohn’s disease. Surg. Clin. N. Am..

[7] Evan A.P., Lingeman J.E., Worcester E.M., Bledsoe S.B., Sommer A.J., Williams J.C., Krambeck A.E., Philips C.L., Coe F.L. (2010). Renal histopathology and crystal deposits in patients with small bowel resection and calcium oxalate stone disease. Kidney Int..

[8] Andersson H., Bosaeus I., Fasth S., Hellberg R., Hultén L. (1987). Cholelithiasis and urolithiasis in Crohn’s disease. Scand. J. Gastroenterol..

[9] Fagagnini S., Heinrich H., Rossel J.-B., Biedermann L., Frei P., Zeitz J., Spalinger M., Battegay E., Zimmerli L., Vavricka S.R. (2017). Risk factors for gallstones and kidney stones in a cohort of patients with inflammatory bowel diseases. PLoS ONE.

[10] Asplin J.R. (2002). Hyperoxaluric calcium nephrolithiasis. Endocrinol. Metab. Clin. N. Am..

[11] Williams H.E., Wandzilak T.R. (1989). Oxalate synthesis, transport and the hyperoxaluric syndromes. J. Urol..

[12] Hesse A., Schneeberger W., Engfeld S., von Unruh G.E., Sauerbruch T. (1999). Intestinal hyperabsorption of oxalate in calcium oxalate stone formers: Application of a new test with [^13^C_2_]oxalate. J. Am. Soc. Nephrol..

[13] Holmes R.P., Goodman H.O., Assimos D.G. (2001). Contribution of dietary oxalate to urinary oxalate excretion. Kidney Int..

[14] Siener R., Ebert D., Nicolay C., Hesse A. (2003). Dietary risk factors for hyperoxaluria in calcium oxalate stone formers. Kidney Int..

[15] Chadwick V.S., Modha K., Dowling R.H. (1973). Mechanism for hyperoxaluria in patients with ileal dysfunction. N. Engl. J. Med..

[16] Dobbins J.W., Binder H.J. (1976). Effect of bile salts and fatty acids on the colonic absorption of oxalate. Gastroenterology.

[17] Witting C., Langman C.B., Assimos D., Baum M.A., Kausz A., Milliner D., Tasian G., Worcester E., Allain M., West M. (2021). Pathophysiology and treatment of enteric hyperoxaluria. Clin. J. Am. Soc. Nephrol..

[18] Earnest D.L., Johnson G., Williams H.E., Admirand W.H. (1974). Hyperoxaluria in patients with ileal resection: An abnormality in dietary oxalate absorption. Gastroenterology.

[19] Nazzal L., Puri S., Goldfarb D.S. (2016). Enteric hyperoxaluria: An important cause of end-stage kidney disease. Nephrol. Dial. Transplant..

[20] Hueppelshaeuser R., von Unruh G.E., Habbig S., Beck B.B., Buderus S., Hesse A., Hoppe B. (2012). Enteric hyperoxaluria, recurrent urolithiasis, and systemic oxalosis in patients with Crohn’s disease. Pediatr. Nephrol..

[21] Hessov I., Hasselblad C., Fasth S., Hultén L. (1983). Magnesium deficiency after ileal resections for Crohn’s disease. Scand. J. Gastroenterol..

[22] Caudarella R., Rizzoli E., Pironi L., Malavolta N., Martelli G., Poggioli G., Gozzetti G., Miglioli M. (1993). Renal stone formation in patients with inflammatory bowel disease. Scanning Microsc..

[23] Best W.R., Becktel J.M., Singleton J.W. (1979). Rederived values of the eight coefficients of the Crohn’s Disease Activity Index (CDAI). Gastroenterology.

[24] Siener R., Pitzer M.S., Speller J., Hesse A. (2023). Risk profile of patients with brushite stone disease and the impact of diet. Nutrients.

[25] Hesse A., Tiselius H.-G., Siener R., Hoppe B. (2009). Urinary Stones: Diagnosis, Treatment, and Prevention of Recurrence.

[26] Tiselius H.-G. (1984). A Simplified estimate of the ion-activity product of calcium phosphate in urine. Eur. Urol..

[27] Tiselius H.-G. (2002). Medical evaluation of nephrolithiasis. Endocrinol. Metab. Clin. N. Am..

[28] Werness P.G., Brown C.M., Smith L.H., Finlayson B. (1985). EQUIL2: A BASIC computer program for the calculation of urinary saturation. J. Urol..

[29] von Unruh G.E., Langer M.A., Paar D.W., Hesse A. (1998). Mass spectrometric-selected ion monitoring assay for an oxalate absorption test applying [^13^C_2_]oxalate. J. Chromatogr. B Biomed. Sci. Appl..

[30] von Unruh G.E., Voss S., Sauerbruch T., Hesse A. (2003). Reference range for gastrointestinal oxalate absorption measured with a standardized [^13^C_2_]oxalate absorption test. J. Urol..

[31] Voss S., Hesse A., Zimmermann D.J., Sauerbruch T., von Unruh G.E. (2006). Intestinal oxalate absorption is higher in idiopathic calcium oxalate stone formers than in healthy controls: Measurements with the [^13^C_2_]oxalate absorption test. J. Urol..

[32] Andersson H., Filipsson S., Hultén L. (1978). Urinary oxalate excretion related to ileocolic surgery in patients with Crohn’s disease. Scand. J. Gastroenterol..

[33] Böhles H., Beifuss O.J., Brandl U., Pichl J., Akçetin Z., Demling L. (1988). Urinary factors of kidney stone formation in patients with Crohn’s disease. Klin. Wochenschr..

[34] Rudman D., Dedonis J.L., Fountain M.T., Chandler J.B., Gerron G.G., Fleming G.A., Kutner M.H. (1980). Hypocitraturia in patients with gastrointestinal malabsorption. N. Engl. J. Med..

[35] Dobbins J.W. (1985). Nephrolithiasis and intestinal disease. J. Clin. Gastroenterol..

[36] Asplin J.R. (2016). The management of patients with enteric hyperoxaluria. Urolithiasis.

[37] Massironi S., Viganò C., Palermo A., Pirola L., Mulinacci G., Allocca M., Peyrin-Biroulet L., Danese S. (2023). Inflammation and malnutrition in inflammatory bowel disease. Lancet Gastroenterol. Hepatol..

[38] Balestrieri P., Ribolsi M., Guarino M.P., Emerenziani S., Altomare A., Cicala M. (2020). Nutritional aspects in inflammatory bowel diseases. Nutrients.

[39] Prieto J.M., Andrade A.R., Magro D.O., Imbrizi M., Nishitokukado I., Ortiz-Agostinho C.L., dos Santos F.M., Luzia L.A., Rondo P.H., Leite A.Z. (2021). Nutritional global status and its impact in Crohn’s disease. J. Can. Assoc. Gastroenterol..

[40] Siener R., Struwe F., Hesse A. (2016). Effect of L-methionine on the risk of phosphate stone formation. Urology.

[41] Asplin J.R. (2022). Neglected analytes in the 24-h urine: Ammonium and sulfate. Curr. Opin. Nephrol. Hypertens..

[42] Ennis J.L., Asplin J.R. (2016). The role of the 24-h urine collection in the management of nephrolithiasis. Int. J. Surg..

[43] Stam S.P., Eisenga M.F., Gomes-Neto A.W., van Londen M., de Meijer V.E., van Beek A.P., Gansevoort R.T., Bakker S.J. (2019). Muscle mass determined from urinary creatinine excretion rate, and muscle performance in renal transplant recipients. J. Cachexia Sarcopenia Muscle.

[44] Ryan E., McNicholas D., Creavin B., Kelly M.E., Walsh T., Beddy D. (2019). Sarcopenia and inflammatory bowel disease: A systematic review. Inflamm. Bowel Dis..

[45] Gelzayd E.A., Breuer R.I., Kirsner J.B. (1968). Nephrolithiasis in inflammatory bowel disease. Am. J. Dig. Dis..

[46] Dobbins J.W., Binder H.J. (1977). Importance of the colon in enteric hyperoxaluria. N. Engl. J. Med..

[47] Hylander E., Jarnum S., Jensen H.J., Thale M. (1978). Enteric hyperoxaluria: Dependence on small intestinal resection, colectomy, and steatorrhoea in chronic inflammatory bowel disease. Scand. J. Gastroenterol..

[48] Peyrin-Biroulet L., Harmsen W.S., Tremaine W.J., Zinsmeister A.R., Sandborn W.J., Loftus E.V. (2016). Cumulative length of bowel resection in a population-based cohort of patients with Crohn’s disease. Clin. Gastroenterol. Hepatol..

[49] Bambach C.P., Robertson W.G., Peacock M., Hill G.L. (1981). Effect of intestinal surgery on the risk of urinary stone formation. Gut.

[50] Siener R., Petzold J., Bitterlich N., Alteheld B., Metzner C. (2013). Determinants of urolithiasis in patients with intestinal fat malabsorption. Urology.

[51] Siener R., Machaka I., Alteheld B., Bitterlich N., Metzner C. (2020). Effect of fat-soluble vitamins A, D, E and K on vitamin status and metabolic profile in patients with fat malabsorption with and without urolithiasis. Nutrients.

[52] Andersson H., Jagenburg R. (1974). Fat-reduced diet in the treatment of hyperoxaluria in patients with ileopathy. Gut.

[53] Kumar R., Ghoshal U.C., Singh G., Mittal R.D. (2004). Infrequency of colonization with *Oxalobacter formigenes* in inflammatory bowel disease: Possible role in renal stone formation. J. Gastroenterol. Hepatol..

[54] Siener R. (2021). Nutrition and kidney stone disease. Nutrients.

[55] Siener R., Seidler A., Voss S., Hesse A. (2016). The oxalate content of fruit and vegetable juices, nectars and drinks. J. Food Compos. Anal..

[56] Voss S., Zimmermann D.J., Hesse A., von Unruh G.E. (2004). The effect of oral administration of calcium and magnesium on intestinal oxalate absorption in humans. Isotopes Environ. Health Stud..

[57] von Unruh G.E., Voss S., Sauerbruch T., Hesse A. (2004). Dependence of oxalate absorption on the daily calcium intake. J. Am. Soc. Nephrol..

[58] Lieske J.C., Lingeman J.E., Ferraro P.M., Wyatt C.M., Tosone C., Kausz A.T., Knauf F. (2022). Randomized placebo-controlled trial of reloxaliase in enteric hyperoxaluria. NEJM Evid..

